# Perigeniculate Giant Cell Tumor of Temporal Bone

**DOI:** 10.7759/cureus.28515

**Published:** 2022-08-28

**Authors:** Eric E Babajanian, Todd C Hollon, Tori A Seasor, William Couldwell, Richard K Gurgel

**Affiliations:** 1 Otolaryngology - Head and Neck Surgery, University of Utah, Salt Lake City, USA; 2 Neurosurgery, University of Utah, Salt Lake City, USA; 3 Pathology, University of Utah, Salt Lake City, USA

**Keywords:** facial nerve palsy, perigeniculate, facial nerve, temporal bone, giant-cell tumor of bone

## Abstract

Giant cell tumors of bone (GCTBs) are benign osteolytic neoplasms that can be treated with either gross-total resection or subtotal resection with adjuvant radiotherapy. For the rare GCTB of the temporal bone, close proximity to critical structures can produce functional deficits and make gross-total resection difficult to achieve without significant morbidity. We present the case of a 28-year-old woman with progressive facial paresis, otalgia, neck pain, imbalance, and subjective hearing loss. She was found to have a facial nerve mass centered at the geniculate ganglion extending into the labyrinthine segment and vestibule. We achieved gross-total resection with preserved facial nerve function as the tumor did not originate from the facial nerve and could be dissected free from the nerve. Final pathology was consistent with GCTB.

## Introduction

Giant cell tumors of bone (GCTBs) are benign osteolytic neoplasms that comprise ~5% of bone tumors; they are most commonly diagnosed in patients aged 20-44 years and demonstrate a slight female predominance (1.2:1) [1,2]. Clinical manifestations include pain, joint effusion, soft-tissue swelling, and a propensity for pathologic fractures at the tumor site [3,4]. These tumors typically present in long bone epiphyseal regions with rare occurrences in the cranial skull [5-12]. Both computed tomography and magnetic resonance imaging modalities are used to characterize GCTBs of the skull. GCTBs can be staged according to either the Enneking staging system, which describes musculoskeletal tumors by their biologic behavior, or the Campanacci system, which is based on radiographic imaging findings [2,13]. GCTBs are treated with surgical excision, with recurrence rates and prognosis correlated with the extent of resection [2]. Depending on the lesion, either gross-total resection (GTR) or subtotal resection (STR) with or without adjuvant radiotherapy can be used [6,12].

GCTBs in the skull most frequently present in the temporal and sphenoid bones but can also rarely involve the frontal, occipital, and parietal bones and the temporomandibular joint [6,14]. Although benign, GCTBs in the skull can be locally aggressive and destructive. Temporal bone involvement may cause symptoms of hearing loss, aural fullness, postauricular pain, tinnitus, and autophony [7,15,16]. Given that the geniculate ganglion of the facial nerve also contains fibers responsible for taste sensation and sensory innervation, a lesion in this area could interfere with both motor and sensory facial nerve components. The close proximity to these critical structures can produce functional deficits and make GTR difficult to achieve without significant morbidity. We present the first published case of a GCTB located in the perigeniculate region of the temporal bone in which GTR was achieved with preservation of facial nerve function.

## Case presentation

**Figure 1:**
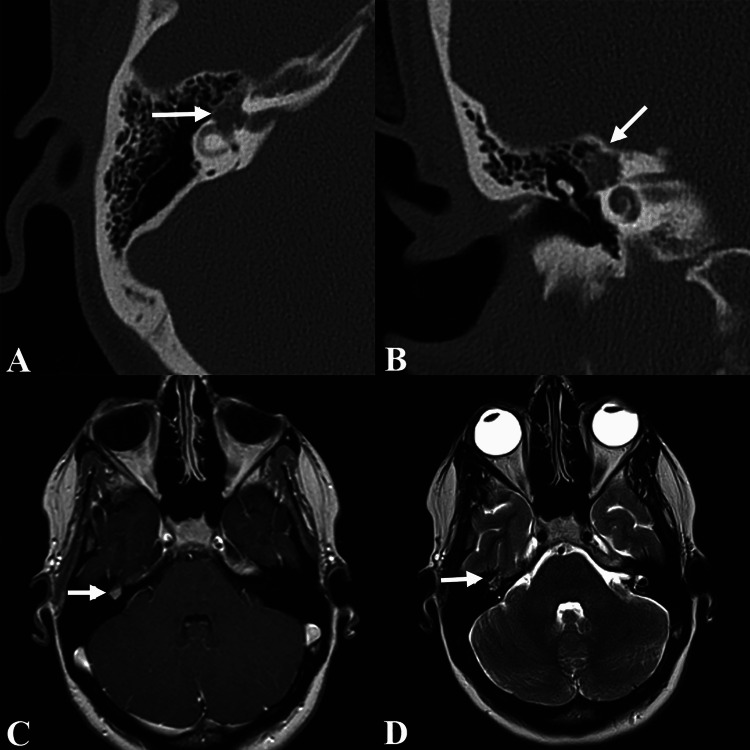
Imaging showing right perigeniculate mass. (A) Axial and (B) coronal computed tomography and (C) axial T1-weighted post-contrast and (D) axial T2-weighted magnetic resonance imaging displaying right perigeniculate mass (arrows).

A 28-year-old previously healthy woman presented with a several-month history of mild imbalance and right-sided progressive facial paresis, otalgia, constant dull neck pain posterior to the mandibular angle, and subjective hearing loss. One month earlier, she had experienced acute complete right-sided facial paralysis. She received treatment at another hospital for presumed Bell’s palsy with prednisone and acyclovir for seven days with significant improvement to mild facial weakness. She denied any significant otologic history. On examination, right-sided facial nerve function was House-Brackmann II/VI with right cheek and lower lip weakness, indicating mild dysfunction. Otologic examination was normal. A 512-Hz tuning fork examination revealed air > bone bilaterally with Weber lateralizing to the right ear. Pure tone average was 15 dB on the right side and 7.5 dB on the left side, with 100% word recognition at 45 dBHL bilaterally. Radiographic imaging revealed a contrast-enhancing, right-sided osteolytic facial nerve mass centered at the geniculate ganglion extending into the labyrinthine segment and vestibule with no apparent calcifications within the mass (Figure 1). This was initially thought to represent a facial neuroma based on imaging characteristics of homogenous enhancement, enlargement of the perigeniculate facial nerve, and lack of calcifications. Management options of close observation with serial imaging versus operative intervention were discussed. Given the risk of progression of her facial paresis, the patient elected to proceed with operative intervention.

The patient underwent resection via a middle fossa craniotomy approach. A mass filled the internal auditory canal just medial to the geniculate ganglion towards the labyrinthine segment of the facial nerve and extended towards the tympanic segment of the facial nerve, initially appearing to originate from the distal vestibular nerve. Upon further dissection, the mass was noted to violate the ampullated end of the superior semicircular canal and extend into the vestibule without a clear site of origin. GTR was performed with resection of the distal vestibular nerve and involved bone, and the facial nerve was anatomically intact and stimulating at the end of the case. Video 1 demonstrates the operative procedure.

**Video 1:** Operative video demonstrating middle cranial fossa approach to gross total resection of a perigeniculate facial nerve mass

**Figure 2:**
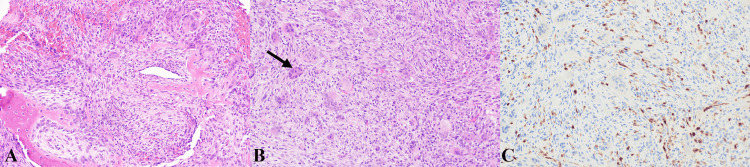
Histopathology slides of mass. (A) Hematoxylin and eosin stain displaying bone trabeculae with osteoblastic rimming surrounded by a spindle cell proliferation with admixed giant cells; (B) hematoxylin and eosin stain displaying mildly atypical, spindle-cell neoplasm with intermixed multinucleated giant cells (arrow); (C) S100 stain demonstrating minimal positive background staining.

Histopathological analysis revealed a spindle-cell neoplasm with mild atypia and intermixed multinucleated giant cells. Trabeculae of bone with osteoblastic rimming were present. Immunohistochemistry demonstrated positive S-100 staining and negative SOX10 and epithelial membrane antigen staining. These findings were consistent with GCTB (Figure 2).

**Figure 3:**
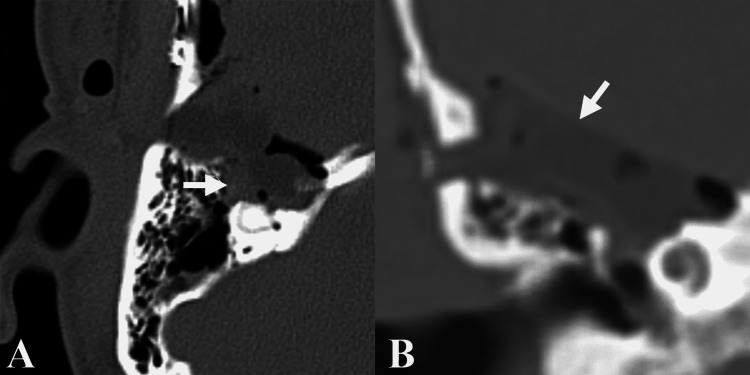
Postoperative imaging demonstrating resection. (A) Axial and (B) coronal computed tomography imaging obtained on postoperative day 1 demonstrating no residual perigeniculate mass; arrows point to fat graft used for reconstruction

Follow-up imaging on postoperative day 1 demonstrated no residual mass (Figure 3). The patient had an unremarkable postoperative course and was discharged home on postoperative day 3. On two-month follow-up, she had mild but improving imbalance and headaches. Neck pain and otalgia had resolved. Facial nerve function was preserved at House-Brackmann II/VI on the right. Postoperative audiogram revealed a right severe mixed hearing loss, with a 40-dB air-bone gap and 56% word recognition at 80 dBHL. Because GTR was achieved, the decision was made to forego adjuvant radiotherapy.

## Discussion

We believe this is the first reported case of GCTB isolated to the perigeniculate area of the temporal bone. Our patient’s presenting symptoms were likely due to a combination of tumor location and mass effect. We were able to preserve facial nerve function because the tumor did not originate from the facial nerve and could be dissected free, leaving the nerve in complete continuity and stimulating at the end of the case. Given the vestibular involvement of the mass, it is surprising that the patient maintains hearing despite GTR.

The decision to proceed with operative intervention for lesions in the perigeniculate area of the temporal bone can be nuanced, as inherent risks of proceeding with either GTR or STR are based on the complex regional anatomy. The transmastoid, translabyrinthine or middle cranial fossa approaches can be used to access the perigeniculate area, with important anatomical structures to include the facial nerve, cochlea, middle ear and semicircular canals. Potential complications specific to surgery of the perigeniculate area can include facial nerve weakness, hearing loss, and dizziness. The decision to proceed with operative intervention should include a shared decision-making approach with consideration of the risks of surgery, clinical deficits at the time of presentation, and potential progression of deficits related to lesion growth. The patient in the present case had presented with ipsilateral facial nerve weakness scored at House-Brackmann II/VI. After discussing options of observation with close follow-up imaging versus operative intervention, the patient elected to proceed with a middle cranial fossa approach to prevent further progression of her facial paresis. 

GCTBs involving the skull are rare, comprising <2% of all GCTBs [17]. Of these, temporal bone lesions comprise ~41% of cranial GCTBs [6]. The current treatment of choice for cranial GCTBs is GTR. Adjuvant radiotherapy is generally recommended for patients who undergo an STR to preserve critical structures [18].

Based on a recent meta-analysis of GCTBs of the skull involving 67 patients, recurrence rates are correlated with extent of resection: GTR has the lowest chance of recurrence (3/34 patients, 8.8%), followed by STR with adjuvant radiotherapy (3/21, 14.3%) and without radiotherapy (7/10, 70%) [6]. In another systematic review evaluating 62 patients with GCTBs of the skull who underwent operative management and had five-year follow-up survival data, all 16 patients who underwent GTR, with or without adjuvant radiotherapy, had recurrence-free survival at five years [12]. In contrast, recurrence-free five-year survival rates were 70.1% (n=33) in patients who underwent STR with adjuvant radiotherapy and 15.4% (n=13) in those who had STR without adjuvant radiotherapy [12].

Studies exploring the efficacy of denosumab found that it has impressive anti-tumoral histologic and radiologic response; however, these studies were conducted on primarily noncranial GCTBs [19,20]. Regardless, denosumab is a promising option for limiting tumoral progression and involvement and thus improving surgical outcomes and morbidity.

## Conclusions

GCTBs of the temporal bone are rare. Given their proximity to critical structures, GCTBs can result in functional deficits including cranial nerve palsies, hearing loss, imbalance, aural fullness, postauricular pain, tinnitus, and autophony. Our case of a GCTB of the perigeniculate region was successfully treated with GTR that preserved facial nerve function. Future studies should evaluate the role of adjuvant therapy in treatment of GCTBs of the temporal bone and lateral skull base.
